# Comparison of Knifelight Surgery versus Conventional Open Surgery in the Treatment of Carpal Tunnel Syndrome

**DOI:** 10.5812/ircmj.4180

**Published:** 2013-05-05

**Authors:** Amin Heidarian, Hamidreza Abbasi, Mehdi Hasanzadeh Hoseinabadi, Azin Hajialibeyg, Seyed Mohammad Kalantar Motamedi, Soroush Seifirad

**Affiliations:** 1 School of Medicine, Hamadan University of Medical Sciences, Hamadan, IR Iran; 2 Endocrinology and Metabolism Research Center, Tehran University of Medical Sciences, Tehran, IR Iran

**Keywords:** Carpal Tunnel Syndrome, Surgical Procedures, Minimally Invasive, Case-Control Studies

## Abstract

**Background:**

A variety of surgical treatment methods for carpal tunnel syndrome are introduced recently, including open surgery, endoscopic and the Knifelight. It is hypothesized that Knifelight method could decrease scar tenderness and time before return to daily activities for patients and is accompanied with less disturbance to fine sensory nerves.

**Objectives:**

To compare the Knifelight instrument and open carpal tunnel release with respect to scar length, operation duration, recovery time needed before return to work and amount of pain three weeks after surgery in patients with neurophysiologically confirmed carpal tunnel syndrome.

**Patients and Methods:**

Fifty nine patients with indication for carpal tunnel release randomly assigned into two groups: open (n=30) or Knifelight (n=29). The patients compared regarding scar length, operation duration, time to return to daily activities and amount of pain at three weeks after operation based on Visual Analog Scale.

**Results:**

There was no significant differences regarding age and sex in the two groups. The scar length, operation duration and time before return to daily activities were significantly lower in the Knifelight group. Although the mean visual analogue scale of Knifelight group found to be lower than the other, it was not statistically significant.

**Conclusions:**

The Knifelight technique is accompanied with advantages over the open surgery regarding operation time, scar length and time to return to daily activities. The pain relieve based on Visual Analog Scale was not statistically different from conventional open surgery.

## 1. Background

Carpal Tunnel Syndrome (CTS) which is first described by Sir James Paget in 1854 is the most common compression neuropathy of upper extremity, with an estimated prevalence of 3.7% in United States (1, 2). Medical treatments of CTS are limited and are not satisfactory in many cases (3, 4). Non-surgical treatment is mainly the splinting and corticosteroid injection which has been effective just in fraction of mild cases (5-7). The surgical treatment has shown better results and more cost effectiveness compared with non-surgical method (8, 9).

Traditionally open release of median nerve was used which seems that it is accompanied by unsatisfactory results due to scar tenderness and pillar pain (10). Cosmetic problems because of large incision in open release method are also important especially in women which are more affected with this problem. Less invasive methods are recently introduced to decrease these unsatisfactory results (10, 11): endoscopic release and Knifelight Carpal tunnel release are among these novel techniques. Results of endoscopic release were not satisfactory. Endoscopic release is expensive and it seems that it is accompanied by a higher prevalence of numbness and paraesthesia (10-13). Results of Knifelight technique which is first reported by Avci et al. were satisfactory (11). Theoretically and with respect to the results of published studies, it seems that this technique decreases scar tenderness due to smaller incision and less disturbance to fine sensory nerves. It is also hypothesized that it could decrease time to take to work for patients (13).

## 2. Objectives

A randomized controlled study was conducted in order to compare Knifelight and open carpal tunnel release results and outcomes. The duration of the procedure, length of the scar at incision site, pain after three weeks and needed time to return to daily activities were compared between groups.

## 3. Materials and Methods

Fifty-nine consecutive patients with CTS who were referred to a tertiary hospital during 2007, enrolled in a randomized controlled trial. All patients had clinical signs or symptoms as well as electro-diagnostic findings consistent with carpal tunnel syndrome and had not responded to nonsurgical management. Patients were randomly divided into 2 groups: thirty subjects were undergone open surgery and 29 patients undergone knifelight procedure. In addition to the type of surgery, the demographic and specific characteristics of all patients recorded by questionnaires at discharge and three weeks after surgery. All fifty-nine subjects were followed up for the specific evaluated parameters consisting scar length (millimeters), procedure duration (minutes), time to return to daily activities as what the patient declares (days), the magnitude of pain at the third week interval after surgery measured by Visual Analog Scale (the score out of 10 declared by patient as the remaining pain compared with the preoperative one).

### 3.1. Surgical Techniques

Open Carpal Tunnel Release and Knifelight Surgery:

Knifelight ® (Stryker, Kalamazoo, MI) is a surgical instrument including a blade which is covered by a forked plastic piece and a light source supplied by a battery which illuminates the plastic forks. After making a small incision on the skin, the site of surgery on flexor retinaculum will be designated by the light (10-12, 14-16).

Following prep and drape and the local or general anesthesia, the arm was compressed by the tourniquet and a towel was placed under the wrist in order to make a 30 to 45 degrees extension. To locate the incision site, a transverse line was drawn at the proximal wrist crease. Second line was drawn between the midpoint of the wrist line (between the scaphoid and hamate bones) and the radial side of the ring finger. Finally the third line was designated transversally along the ulnar side of fully abducted thumb. A 1 to 1.5 cm incision was made along the second line proximal to the intersection with the third line. The incision site has less subcutaneous fat tissue compared with the open surgery. Using the Freer elevator to approach proximally under the fat and along with the ulnar edge of palmaris longus tendon, it reaches space between interthenar fascia and flexor retinaculum. A 3 to 4 mm incision was made at the distal edge of flexor retinaculum close to the ulnar side and then the Knifelight was turned on and operation room was darkened as much as possible. The Knifelight was inserted through the incision as the longer tip was under flexor retinaculum and short tip placed over it in the space between interthenar fascia and flexor retinaculum. At this time, only the light spot of the short tip was visible clearly through the skin. The desired site was determined with the light source and median nerve was released from the carpal tunnel compression. The device was pushed gently to the proximal until the blade was cut the flexor retinaculum. Then it was pulled back slightly to overview the cutting. This process was repeated until the retinaculum was released completely. In this stage, light spots at both tips of the device were visible through the skin. The Knifelight was taken out and the tourniquet was unfastened to evaluate hemostasis. The palmar fascia was stitched with absorbable suture and skin was closed with 2.0 or 3.0 nylon suture. Finally a pressure dressing was applied in place. The patients were encouraged to start daily activities after a week and sutures were removed after 10 to 14 days (10-12).

### 3.2. Statistical Analysis

All analyses were done utilizing SPSS version 10 (SPSS Inc. Chicago, IL, USA). Sex distribution was tested via Chi-squared test. Age, Operation duration, scar length, time until return to daily activity and visual analog scale were compared between two groups using t-test. Statistical significance for all tests was set at 5 percent level.

### 3.3. Ethical aspects

The risks and objectives of the study were explained to all the participants and written informed consent was obtained. The study protocol was reviewed and approved by ethics committee of Hamadan University of Medical Sciences.

## 4. Results

Mean age of the patients with CTS in groups of open and Knifelight surgery was 45.5 and 49.6 years respectively. There was no significant difference in the age of two groups (P value = 0.86). The subjects were also well distributed randomly in the two groups regarding sex. Women were 93.3% and 86.2% of patients in open and Knifelight groups respectively (Table 1).

**Table 1.** Basic Demographic Data of two Different Groups of Carpal Tunnel Release Surgery

DemographicInformation	Open Surgery	Knifelight	P value
**Mean Age (years)**	45.5±10.2 (41.7-49.3)^a^	49.7± 7.9 (46.7-52.7)	0.86
**Female Population (%)**	93.3	86.2	0.6

^a^Mean ± SD (95% lower-upper Confidence Interval)

Table 2 shows the summary of main results. Mean duration of surgery was 12.5 minutes longer for open surgery than that of Knifelight (P value < 0.001). We also compared the length of scar in both techniques in millimeters, which significantly showed more than 60 percent shorter scar length for incisions made in Knifelight technique.

**Table 2.** The Main Results of Open Surgery Compared With theKnifelight

	Open Surgery	Knifelight	P value
**Mean Operation Duration (minutes)**	21 ± 8.9 (17.7-24.4)^a^	8.5 ± 4.2 (6.9-10.1)	0.000
**Mean Scar Length (millimeters)**	40.7 ± 5.6 (38.6-42.8)	14.8 ± 3.7 (13.4-16.2)	0.000
**Mean time until return to daily activity (days)**	51.9 ± 31.0 (40.3-63.5)	34.4 ± 21.8 (26.1-42.7)	0.015
**Pain at 3w interval (Visual Analog Scale)**	1.80 ± 1.58 (1.2-2.4)	1.38 ± 1.08 (1-1.8)	0.24

^a^Mean ± SD (95% lower-upper Confidence Interval)

Compared to the conventional open surgery, Knifelight significantly decreased the time needed before return to daily activities (P value = 0.015). The patients who were underwent open surgery became active after 51.8 days which was 50% longer than the time needed for the counterparts of Knifelight group. The open surgery resulted in return to daily activities after mean recovery period of 51.8 days which was 50% longer than the time needed for the patients who were underwent Knifelight surgery (Table 2). Although the mean visual analogue scale of Knifelight group found to be lower than the other, it was not statistically significant (P value = 0.24).

## 5. Discussion

According to the results of our study, compared to the conventional open surgery, Knifelight technique significantly decreased the time to return to work. This is in accordance with Helm et al study results (12). Results of Bhattacharya et al. study showed no significant decrease in return to work. Although these authors used "Weeks" for the return to work period, using days may result in significant results. This phenomenon called "significance by chance" in statistics. It is better to define a definite clinical and practical scaling method for time to return to work period in order to avoid such statistical difference in results and interpretation of different studies. Knifelight scars were significantly shorter than open surgery scars in our study. A smaller incision, which is not beyond the weight bearing part of the hand, could decrease complications and is very important for cosmetic aspects of surgery (10).

The mean duration of surgery was significantly lower in Knifelight method in our study. While the mean visual analogue scale of Knifelight group found to be lower than the other, it was not statistically significant. Although Yeo et al reported no difference between two methods of open and Knifelight in regard of pain improvement, numbness and patient satisfaction, results of most of other studies showed significant deference in scar tenderness after Knifelight method compared to open release (10-12, 16). Small sample size; impossibility of blinding patients and care providers which may have caused bias in our study results were among our main study limitations. However the latter bias is inevitable.

Covariate adaptive randomization was the method of choice for randomization process of this study, however it was not applied and simple randomization method was used for randomization. In conclusion according to the results of this study, compared to the open release method, Knifelight technique could significantly decrease the mean duration of surgery, incision length and time to return to work.
